# U–Pb ages of detrital zircons in Cretaceous–Paleogene/Neogene kaolins within Eastern Dahomey and Niger Delta Basins (Nigeria) as provenance indicators

**DOI:** 10.1038/s41598-021-93453-9

**Published:** 2021-07-05

**Authors:** Olaonipekun Oyebanjo, Georges-Ivo Ekosse, John Odiyo

**Affiliations:** 1 Natural History Museum, Obafemi Awolowo University, Ile-Ife, Osun State Nigeria; 2 Directorate of Research and Innovation, University of Venda, P/Bag X5050, Thohoyandou, 0950 South Africa; 3 School of Environmental Sciences, University of Venda, P/Bag X5050, Thohoyandou, 0950 South Africa

**Keywords:** Environmental sciences, Solid Earth sciences

## Abstract

Detrital zircon grains within four (4) deposits including two (2) Cretaceous and two (2) Paleogene/Neogene kaolins in Nigeria were analysed using U–Pb LA-SFICP-MS to determine their provenance. The zircon textures were dominated by xenocrystic cores and oscillatory zoning in the Cretaceous and Paleogene/Neogene kaolins, respectively. The Th/U ratios obtained for the detrital zircon grains within the kaolins were predominantly within known values for rocks with magmatic origin. The age populations obtained for the detrital zircon grains were dominated by values from 529 to 978 Ma within the Neoproterozoic, followed by values from 1754 to 2497 Ma of the Paleoproterozoic. Detrital zircon ages obtained between 553.2 ± 6.2 and 583.5 ± 2.0 Ma represent part of the minimum provenance ages for the primary minerals that were kaolinised. The Cretaceous–Paleogene/Neogene kaolins were derived from parent rocks of Eburnean and Pan African ages within the Western and Northern Nigeria Basements.

## Introduction

Age constraints for kaolins have relied greatly on relative dating by identifying localities of deposits where they are correlated to profiles developed on or overlain by sediment or rock of known ages^1,2^. Absolute geochronological information using conventional radiometric techniques in dating the timing of kaolinisation is currently a major problem in geochronology. This is so because suitable radiogenic isotope systems such as K, Rb, or U are highly depleted in kaolins^3^. Several attempts have been made outside Nigeria using K–Ar and Rb–Sr dating techniques which have yielded ambiguous dates, making geoscientists skeptical to accept these ages as possible timing of kaolinisation^4^. Detrital zircons can be linked to possible bedrock source regions by their ages to constrain the depositional ages and provenance of sediments. In active tectonic settings, they commonly provide new age constraints on the depositional age of the host rocks, which can be no older than the youngest concordant zircon^5^.

Most studies on zircon geochronology in Nigeria have been directed towards the determination of the ages of the crystalline basement rocks^6–11^. Despite the large occurrences of kaolin deposits and studies on kaolins in Nigeria, no investigation on the provenance of the Cretaceous and Paleogene/Neogene kaolins using detrital zircon geochronology has been reported. Stable isotope data of the Cretaceous–Paleogene/Neogene kaolins indicated that the kaolins were sedimentary in origin (detrital) because of the denudation of deeply weathered crystalline basement rocks before transportation and deposition^12^. Considering that the African and South American continents were previously joined with common geologic history until their breakup into separate continents during the Cretaceous period and the vast occurrences of kaolins in the two continents, the resulting baseline geochronological information from this study will be useful in evaluating possible linkages between these continents. Hence, this study investigated the U–Pb detrital zircon geochronology of kaolins from the Eastern Dahomey and Niger Delta Basins in Nigeria. The obtained results were used to deduce the possible sources of sediments that formed the kaolins.

## Geologic background

**Figure 1:**
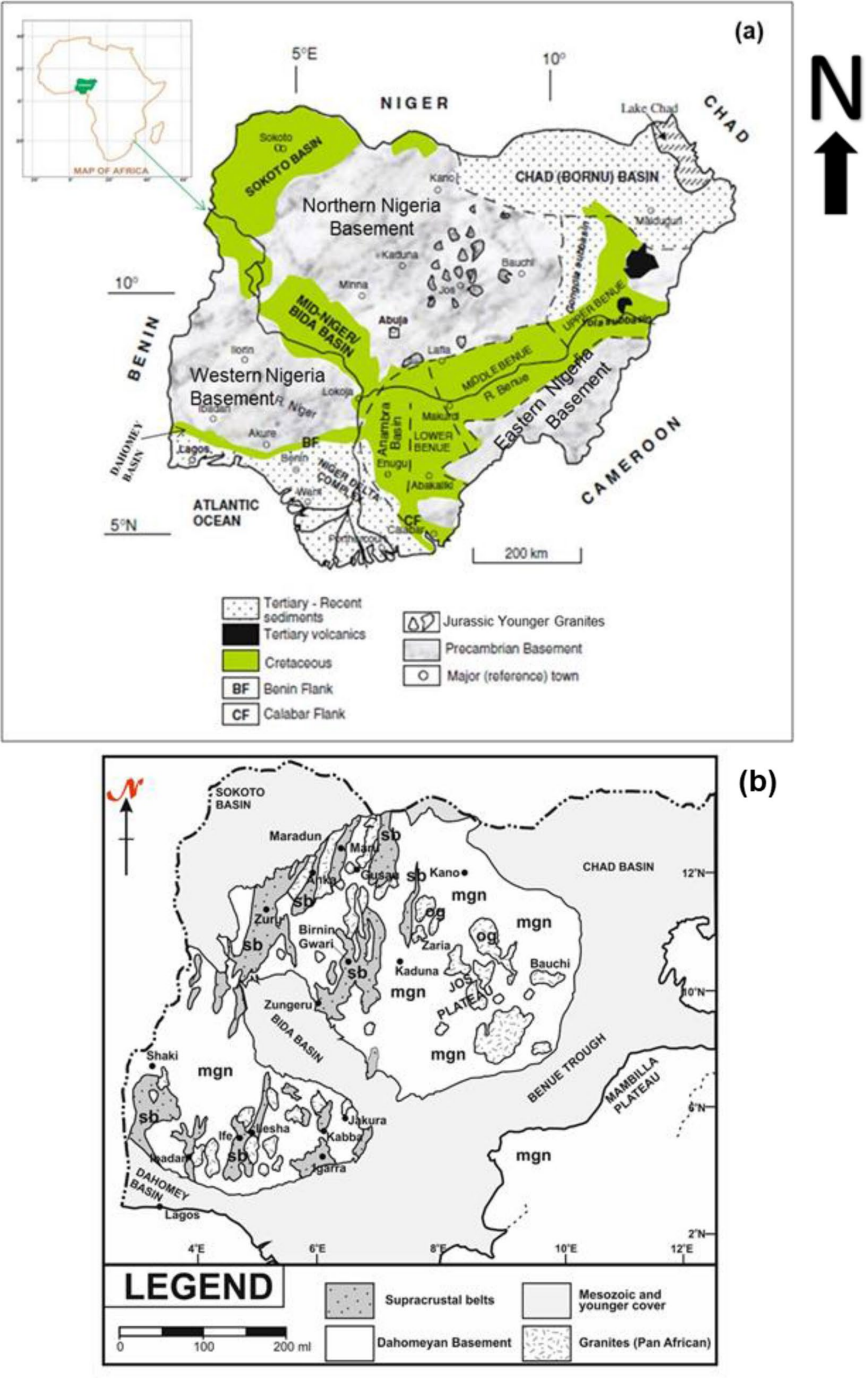
(**a**) Geologic Map of Nigeria showing the Basement complex and the sedimentary basins (Modified after^13^) and (**b**) Basement Geology of Nigeria: The migmatite-gneiss complex (mgn), the schist belts (sb) and the older granites (og) (Modified from^15^).

The geology of Nigeria consists of the Precambrian crystalline Basement Complex (> 600 Ma), Jurassic younger granites (200–145 Ma), and the Cretaceous—Recent sediments (< 145 Ma) (Fig. 1a). The Basement Complex covers about 50% of the total landmass and has strong structural influence on the architecture and evolution of the sedimentary basins^13^. The Basement Complex is believed to have experienced a rugged tectonic cycle of formation with four (4) orogenies, the Liberian (2700–2500 Ma); the Eburnean (2500–2000 Ma); the Kibaran (2000–1100 Ma); and the Pan African (750–450 Ma)^14^. The Basement Complex of Nigeria constitutes part of upper Proterozoic mobile belts sandwiched between West African and Congo-Kasai Cratons. Obaje^13^ recognised four (4) main petrological groups within the Nigeria Basement Complex: the migmatite-gneiss-quartzite complex (MGQC), the schist belts, the older granites, and the undeformed acid and basic dykes (Fig. 1b).

**Figure 2:**
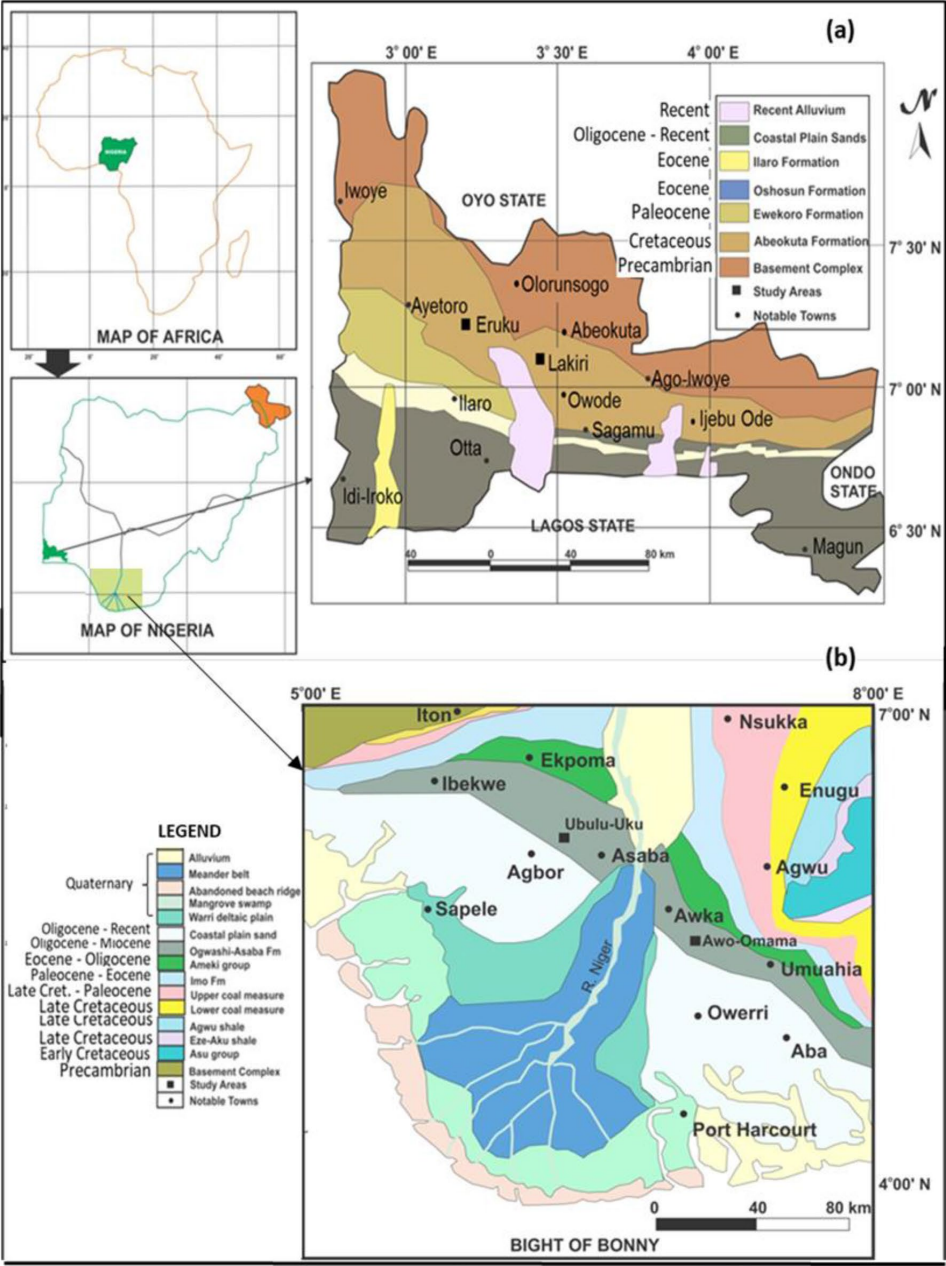
Geologic maps of (**a**) Eastern Dahomey and (**b**) Niger Delta Basins showing the study areas (Modified after^16^).

**Figure 3:**
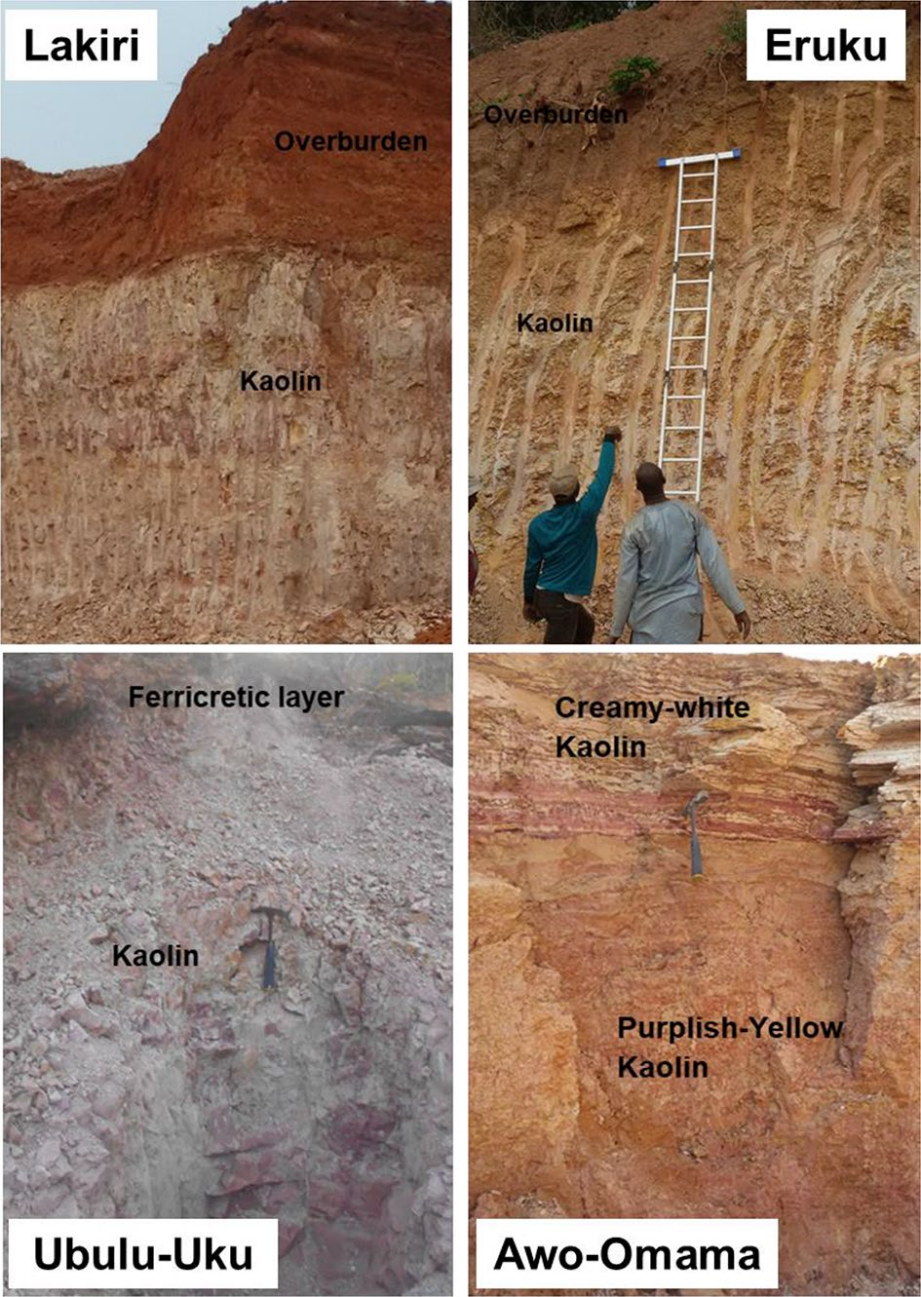
Profile views of the studied Cretaceous–Paleogene/Neogene kaolin deposits.

The studied Cretaceous Eruku and Lakiri kaolin deposits occur within the Abeokuta Group (Ise, Afowo, and Araromi Formations) of the Eastern Dahomey Basin, whereas the Paleogene/Neogene Awo-Omama and Ubulu-Uku kaolin deposits occur within the Ogwashi-Asaba Formation of the Niger Delta Basin (Fig. 2). The profile views of each of the kaolin deposits are presented in Fig. 3. Tables 1 and 2 summarises the stratigraphy and lithologic descriptions of the formations within the Eastern Dahomey and Niger Delta Basins as described by Nwajide^16^. More detailed description on the geology and stratigraphy of the Basins have been discussed by Oyebanjo et al.^12,17,18^. The mineralogical analyses of the kaolins showed that the Lakiri deposit is composed of pure kaolins, whereas the Eruku, Awo-Omama, and Ubulu-Uku deposits were composed of sandy kaolins. This is due to the relatively higher percentages of quartz in the latter deposits^19^. Hence, the kaolins were mainly composed of kaolinite and quartz with trace amounts of muscovite, anatase, hematite, and goethite.

**Table 1:** Summary of formations within the Eastern Dahomey Basin (Modified from^16^).

Geologic age (Ma)	Formation	Lithology
Recent (0.01–0)		Alluvium
Oligocene–Pleistocene (5.3–0.01)		Coastal plain sands
Middle-Upper Miocene (15.9–5.3)	Ilaro	Sands, phosphatic beds with intercalation of shales and clays
Middle Eocene (38.0–41.3)	Oshosun	Mudstones, claystones with interbeds of pebbly sandstones
Lower-Middle Eocene (41.3–56.2)	Akinbo	Shale with silty, glauconitic marl and conglomerate
Upper Paleocene (56.2–59.2)	Ewekoro	Thinly bedded glauconitic and sandy limestones
Maastrichtian–Paleocene (65–59.2)	Araromi	Sands underlain with shale and siltstones with thin interbeds of limestones and marls
Turonian- Maastrichtian (95–65)	Afowo	Sandstones with interbeds of shales, siltstones, and clays
Valanginian–Barremian (140–125)	Ise	Gritty sands and siltstones with interbeds of kaolinitic clays
Precambrian basement complex

**Table 2:** Summary of surface and subsurface formations within the Niger Delta (After^16^).

Geologic age (Ma)	Surface (outcrop) formations	Lithology	Subsurface formations	Lithology
Oligocene–Present (28–0)	Benin	Cross-bedded sands with clay lenses and lignites	Benin	Sands
Oligocene–Miocene (28.1–5.3)	Ogwashi-Asaba	Clays, silts, sands and thin to thick lignite seams	Agbada	Alternating sands/mudrock
Eocene–Early Oligocene (47.8–28.1)	Ameki	Clays and silts with thin shelly Limestones		
Paleocene–Early Eocene (66–47.8)	Imo	Shales with sand lenses, marls and fossiliferous limestones	Akata	Marine shales with sandy and silty beds

## Analytical methods

### Zircon separation

Zircon crystals from four composite samples representing each of the kaolin deposits collected from Eruku, Lakiri, Ubulu-Uku and Awo-Omama were analysed at the Central Analytical Facility (CAF), Stellenbosch University (SU), South Africa. Samples (2–5 kg) were crushed and sieved through a 350 μm sieve, followed by panning and magnetic separation using a Frantz Isodynamic Separator based on the intensity of current generated. The non-magnetic fraction was further separated by a heavy liquid technique, using 99% Methyleniodide with a density of 3.325 g/cm^3^. After drying of the heavy mineral fraction, about 120 zircon grains were randomly handpicked, using a Wild M3Z microscope.

### Field emission scanning electron microscopy imaging

Zircon grains were mounted in an epoxy mould that was ground down and then polished using a 3-micron pad followed by 1-micron pad to finish and a 1-micron diamond paste on a Struers Rotopol-35 equipment to expose grain interior. The mould was gold coated using an Edwards S150A sputter coater. Cathodoluminescence (CL) and backscatter (BS) images were obtained with a Zeiss MERLIN Field Emission Scanning Electron Microscope (FE-SEM). The CL images were used to identify different internal zoning patterns within the individual zircon grains (core and rim); whereas the BSE images were used to constrain the ablated spots to avoid parts with fractures and holes which could affect the quality of results.

### U–Pb laser ablation—single collector—magnetic sectorfield—inductively coupled plasma—mass spectrometry: data capture and treatment

The U–Pb zircon isotopic analyses with a spot diameter of 30 μm and a crater depth of approximately 15 to 20 μm following the methods described by Gerdes and Zeh^20^ and Frei and Gerdes^21^ were performed. A Thermo Finnigan Element2 mass spectrometer coupled to a NewWave UP213 laser ablation system at the Central Analytical Facility (CAF), Stellenbosch University (SU), South Africa was used. The instrument’s operating conditions are summarised in Table 3.

**Table 3:** Instrument specifications for the LA-SF-ICP-MS operating conditions.

**Laser ablation system**	
Make, Model & type	ESI/New Wave Research, UP213, Nd:YAG
Ablation cell & volume	Custom build low volume cell, volume ca.3 cm^3^
Laser wavelength	213 nm
Pulse width	3 ns
Fluence	2.5 J/cm^−2^
Repetition rate	9 Hz
Spot size	30 μm
Sampling mode/pattern	30 μm single spot analyses
Carrier gas	100% He, Ar make-up gas combined with T-connector close to sample cell
Pre-ablation laser warm-up (background collection)	21 s
Ablation duration	21 s
Wash-out delay	20 s
Cell carrier gas flow	0.35 l/min He
**ICP-MS instrument**	
Make, model & type	Thermo Finnigan Element 2 single collector HR-SF-ICP-MS
Sample introduction	Via conventional tubing
RF power	1100 W
Make-up gas flow	1.0 l/min Ar
Detection system	Single collector secondary electron multiplier
Masses measured	202, 204, 206, 207, 208, 232, 233, 235, 238
Integration time per peak	4 ms
Total integration time per reading	1 s (represents the time resolution of the data)
Sensitvity	20,000 cps/ppm Pb
Dead time	6 ns

Mass ^202^Hg was measured to monitor the ^204^Hg interference on ^204^Pb (using a ^202^Hg/^204^Hg-ratio of 4.36), which was typically ∼ 200–400 cps. If necessary, common Pb corrections were done using the interference and background-corrected ^204^Pb signal in combination with a model Pb composition^22^. A common Pb correction was necessary if the corrected ^207^Pb/^206^Pb was smaller and significantly different from the uncorrected ^207^Pb/^206^Pb (e.g., lay outside of the internal errors of the uncorrected ^207^Pb/^206^Pb ratio)^21^. The combination of Iolite v. 3.1 and VisualAge software were used in data processing and reduction^23,24^. For quality control, data normalisation to zircon reference materials such as GJ1 (602 ± 1 Ma), Plešovice (337 ± 1 Ma), and M127 (524 ± 1 Ma), respectively^25–27^ was also conducted. The calculation of concordia ages, and plotting of concordia diagrams, was performed using Isoplot/Ex 3.0^28^. The concordance ((age ^206^Pb/^238^U)/(age ^207^Pb/^206^Pb) × 100) was calculated as described by Verma et al.^29^. For interpretation of the detrital zircon age data, only concordant or nearly concordant (≤ 10% discordant) data were considered. Ages < 1300 Ma and > 1300 Ma were obtained from the ^206^Pb/^238^U and ^207^Pb/^206^Pb ratios, respectively. Possible Pb loss causes lesser amounts of ^207^Pb and hence, ^207^Pb/^206^Pb ages gradually becomes inaccurate for ages < 1300 Ma^30,31^. The complete U–Pb dataset is provided in Appendix A as Supplementary Data.

## Results

### Zircon morphology, internal textures and geochemistry

**Figure 4:**
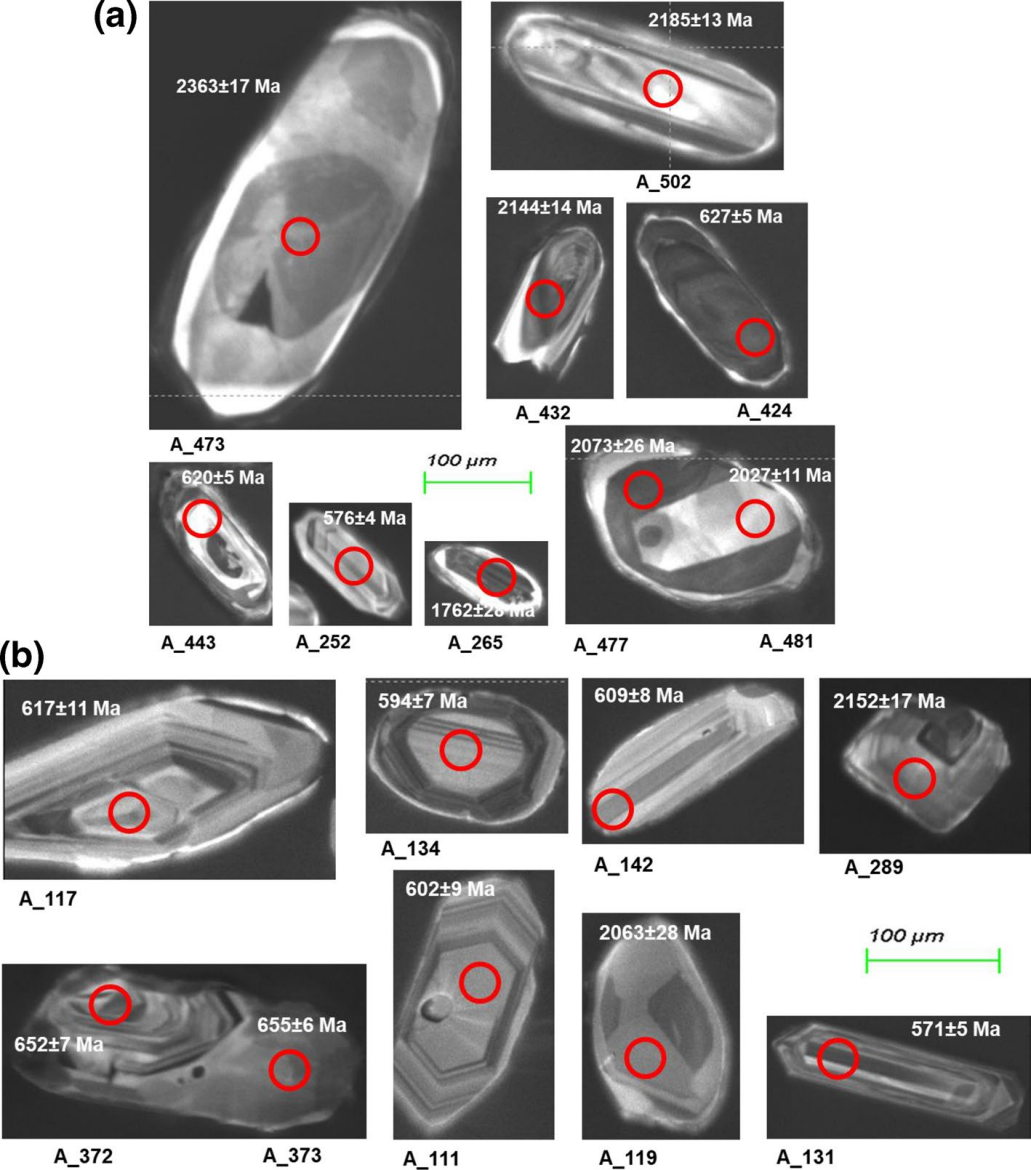
(**a**) CL images of selected zircon grains in the studied Cretaceous kaolins. Red circles indicate the spot location. (**b**) CL images of selected zircon grains in the studied Paleogene/Neogene kaolins. Red circles indicate the spot location.

The detrital zircon grains from the Cretaceous Lakiri kaolin deposit were subhedral with a few euhedral and fragmented crystals. The grains were predominantly subrounded to rounded with few needle-like crystals. The sizes of the zircons ranged between 70 and 300 μm. The zircon internal textures were dominated by xenocrystic cores with rims followed by homogeneous unzoned cores. Few sector and chaotic zoning were observed. Oscillatory zonings were not too common (Fig. 4a). The needle-like acicular zircon crystals are suggestive of rapid crystallization, whereas xenocrystic cores themselves do not yield useful clues as to their origin^32^. Most of the Cretaceous Eruku detrital zircon grains were euhedral to subhedral with a few rounded grains. The sizes of the zircons ranged between 50 and 200 μm. The zircon textures were dominated by xenocrystic cores with some having oscillatory zones followed by homogeneous unzoned cores (Fig. 4a).

Detrital zircon grains from the Paleogene/Neogene Awo-Omama deposit were euhedral with few subhedral to subrounded crystals. The sizes of the zircons ranged between 60 and 200 μm. The zircon textures were dominated by oscillatory zoning followed by those with xenocrystic cores (Fig. 4b). Most of the detrital zircon grains from Paleogene/Neogene Ubulu-Uku kaolin deposit were euhedral with few subhedral to subrounded and rounded crystals. The sizes of the zircons ranged between 50 and 200 μm. The zircon textures were dominated by oscillator zoning followed by those with xenocrystic cores (Fig. 4b). Few grains showed sector zonings. Oscillatory zoning is considered as evidence of magmatic origin^32^.

The dominance of euhedral grains suggests little sedimentary transport, whereas the dominance of rounded grains suggests input of materials that underwent prolonged and possibly multicycle transport^33^. The xenocrystic cores had varying colour domains (lighter or darker) depending on the differences in the U-content.

**Figure 5:**
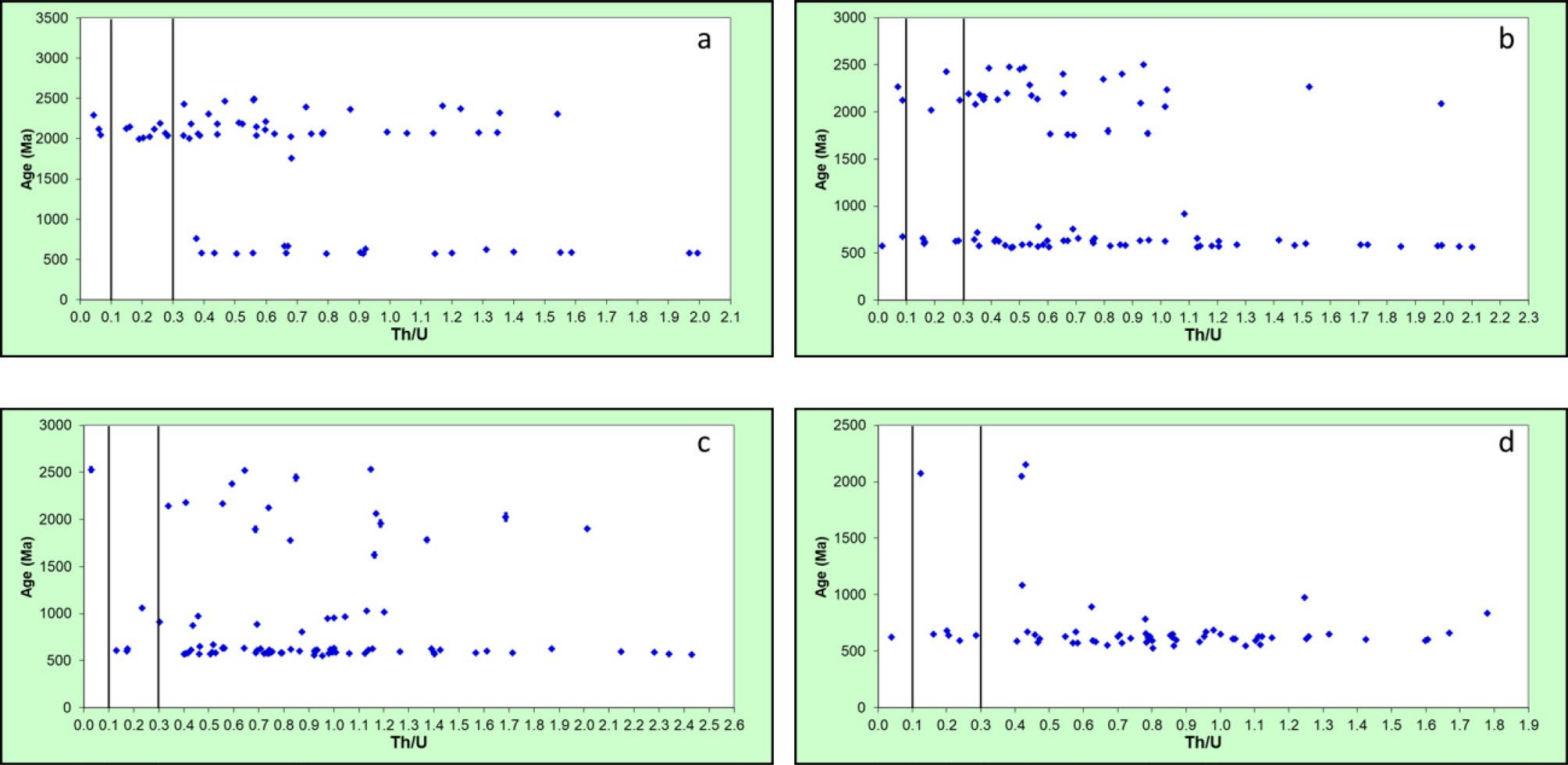
Th/U variation with age (Ma) in the studied (**a**) Lakiri; (**b**) Eruku; (**c**) Awo-Omama; and (**d**) Ubulu-Uku kaolins. The 0.1 and 0.3 black lines mark the upper and lower limits of zircon of metamorphic or magmatic origin, respectively^35^.

The Th/U ratios of the studied detrital zircon grains ranged from 0.01 to 2.43, though most zircons had ratios > 0.3 (Fig. 5). Three groups of zircon origins based on the Th/U ratios have been identified^34–36^. Th/U > 0.3, 0.3–0.1, and < 0.1 represent zircons of igneous origin, igneous or metamorphic origin, and metamorphic origin, respectively. The Th/U ratios obtained for the detrital zircon grains within the Cretaceous and Paleogene/Neogene kaolins were suggestive of predominantly magmatic in origin. There are exceptions to this classification. For instance, in high-temperature and ultrahigh-temperature metamorphic rocks, Th/U ratio is frequently > 0.1^37^. Hence, it is a rule of thumb which requires additional evidence from petrological, chemical, and structural observations to substantiate the inferences^38^.

### U–Pb detrital ages

Most analyses of the zircon grains in each of the kaolin deposits were located in either/both the core and rim region of the grains. Analyses plotting away (above/below) from the concordia (line connecting equal ages) are believed to have lost common lead (^204^Pb). The age frequency distribution for the zircon ages with ≤ 10% discordance was plotted on the probability density plots by Isoplot function.

**Figure 6:**
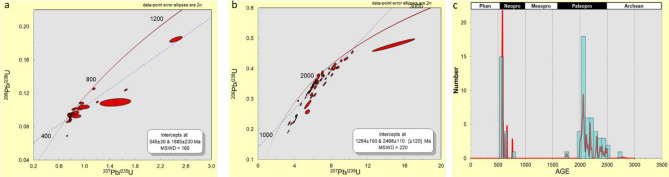
U–Pb concordia diagram of detrital zircon grains (**a**) Ages < 1300 Ma, (**b**) Ages > 1300 Ma, and (**c**) probability density diagram of detrital zircon ages with concordance between 90 and 110% in the studied Lakiri kaolins.

A total of 101 analyses were undertaken for the Lakiri kaolins and are shown on concordia plots (Fig. 6a,b), which displayed clusters around ca. 570 and 2000 Ma. These results showed that 35% of the analyses had > 10% discordance. The Neoproterozoic zircons had ages between ca. 570 and 763 Ma (20 grains), whereas zircon ages within the Paleoproterozoic ranged from ca. 1756 to 2497 Ma (45 grains). A single zircon grain with ca. 2769 Ma was identified within the Archean (Fig. 6c).

**Figure 7:**
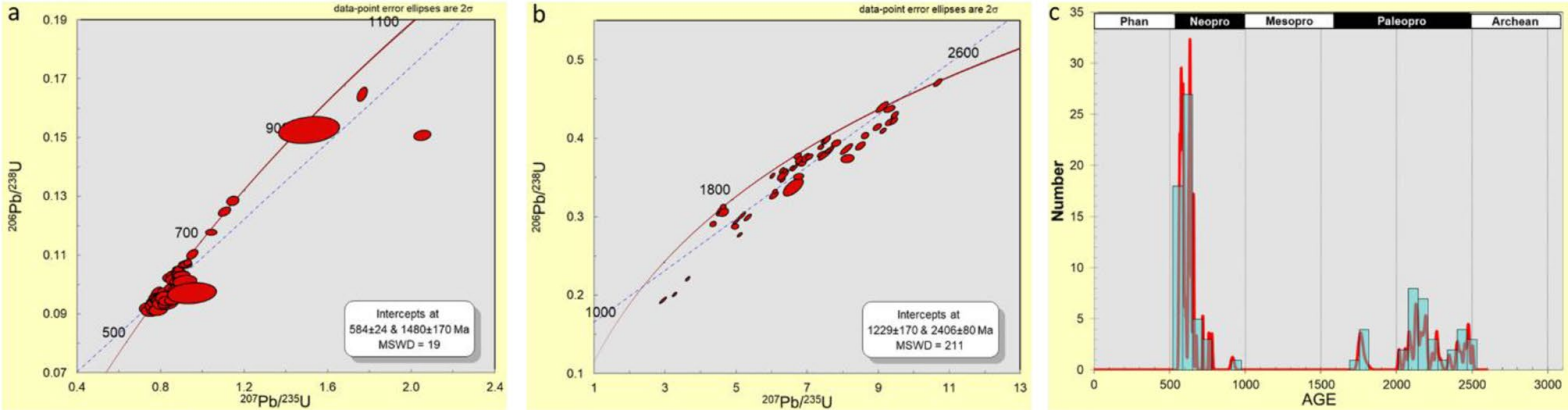
U–Pb concordia diagram of detrital zircon grains (**a**) Ages < 1300 Ma, (**b**) Ages > 1300 Ma, and (**c**) probability density diagram of detrital zircon ages with concordance between 90 and 110% in the studied Eruku kaolins.

The results of the 114 analyses of zircon grains from Eruku kaolins showed that 22% had > 10% discordance with clusters around ca. 580 and 1750 Ma (Fig. 7a,b). Fifty-four zircon ages within the Neoproterozoic (ca. 560–915 Ma), thirty-four zircon ages within the Paleoproterozoic (ca. 1754–2480 Ma), and one Archean age (ca. 2503 Ma) were obtained (Fig. 7c).

**Figure 8:**
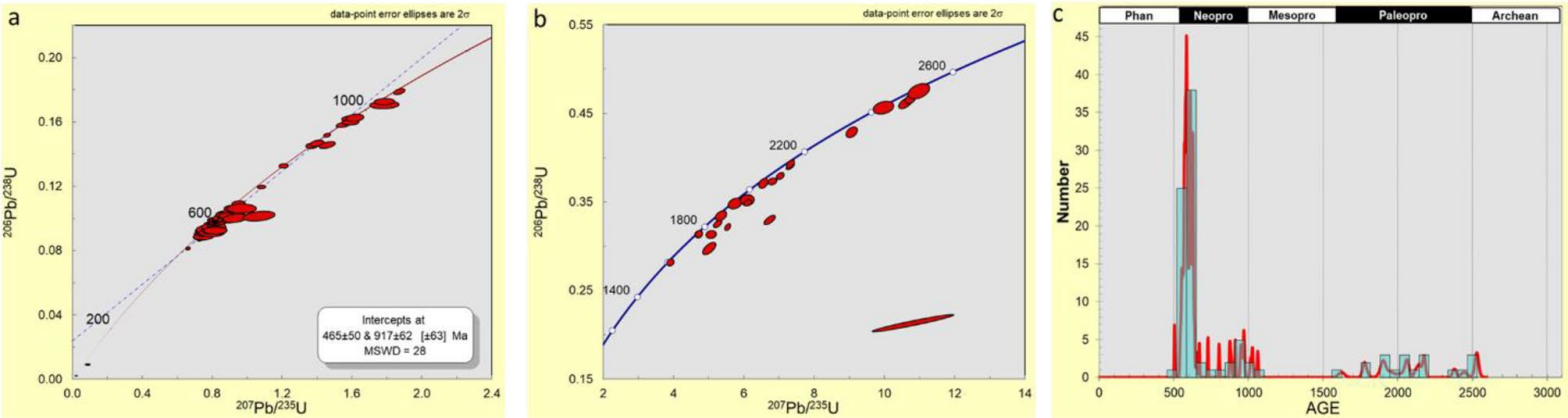
U–Pb concordia diagram of detrital zircon grains (**a**) Ages < 1300 Ma, (**b**) Ages > 1300 Ma, and (**c**) probability density diagram of detrital zircon ages with concordance between 90 and 110% in the studied Awo-Omama kaolins.

A relatively big cluster around ca. 580 Ma and smaller clusters around ca. 800 and 900 Ma were observed on the concordia plots for the 112 zircon analyses for the Awo-Omama kaolins (Fig. 8a,b), with 15% having > 10% discordance. The Neoproterozoic zircons had ages between ca. 552 and 972 Ma (20 grains), whereas zircon ages within the Paleoproterozoic ranged from ca. 1016 to 2445 Ma (45 grains). Three zircon grains with ages between ca. 2524 and 2534 Ma were identified within the Archean (Fig. 8c).

**Figure 9:**
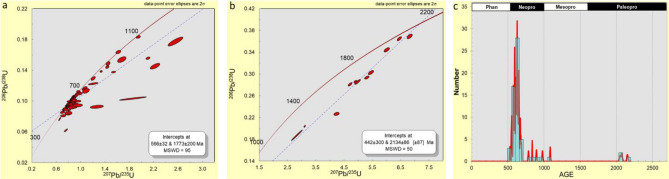
U–Pb concordia diagram of detrital zircon grains (**a**) Ages < 1300 Ma, (**b**) Ages > 1300 Ma, and (**c**) probability density diagram of detrital zircon ages with concordance between 90 and 110% in the studied Ubulu-Uku kaolins.

The concordia plots (Fig. 9a,b) showed a cluster around ca. 570 Ma for the 114 zircon minerals separated from Ubulu-Uku kaolins. The results showed that 47% of the analyses had > 10% discordance. Most of the analysed zircon grains yield Neoproterozoic ages with values between ca. 529–978 Ma (57 grains). Paleoproterozoic ages between ca. 2051 and 2152 Ma were also obtained for three grains (Fig. 9c).

## Discussion

### Implications for Provenance of the Cretaceous–Paleogene/Neogene kaolins

The minimum provenance ages of the rocks from which the studied kaolins were derived must be the age of the youngest detrital zircon, provided no disturbance occurred in the U–Pb isotopic system^39,40^. In obtaining the youngest age^41,42^, the weighted average (WA) age of the youngest zircon population group was used. The determined minimum provenance age estimates for the Cretaceous (579.7 ± 2.3 Ma for Lakiri and 577.9 ± 2.8 Ma for Eruku) and Paleogene/Neogene (583.5 ± 2 Ma for Awo-Omama and 553.2 ± 6.3 Ma for Ubulu-Uku) kaolins in the Eastern Dahomey and Niger Delta Basins in Nigeria correspond to the Ediacaran Period (645–541 Ma) of the Neoproterozoic Era (1000–541 Ma).

U–Pb ages of detrital zircon grains present within the Cretaceous–Paleogene/Neogene kaolins can be used to identify specific sediment sources. A detailed inspection of the probability density plots revealed that the ages in the range of 1000–541 Ma (Neoproterozoic Era) constituted 48% and 85% of the Cretaceous and Paleogene/Neogene kaolins, respectively. Ages within 2500–1600 Ma (Paleoproterozoic Era) constituted 52% and 13% of the Cretaceous and Paleogene/Neogene detrital zircons, respectively. Whereas the Paleogene/Neogene kaolins (particularly Awo-Omama kaolins) had 4% of ages within 4000–2500 Ma (Archean). The ages obtained for the detrital zircons within the studied Cretaceous–Paleogene/Neogene kaolins reflect major inputs of zircon grains from predominantly the Eburnean and Pan African orogenies with little contributions from the Liberian and Kibaran orogenies (Table 4).

**Table 4:** Percentages of U–Pb ages of detrital zircon grains (using ≤ 10% discordance) within the studied Cretaceous–Paleogene/Neogene kaolins.

Orogeny*	Ages (Ma)*	Nigeria				Brazil	
		Lakiri (n = 68)	Eruku (n = 89)	Awo-Omama (n = 97)	Ubulu-Uku (n = 61)	Soft Capim River** (n = 30)	
Liberian (Archean)	2700–2500	2%	–	4%	–	10%	
Eburnean (Mid–Late Paleoproterozoic)	–	67%	34%	10%	5%	50%	
Kibaran (Early Mesoproterozoic–Mid Paleoproterozoic)	2000–1100	3%	6%	8%	–	37%	
Pan African	750–450	28%	60%	78%	95%	3%	

*^14^; **^43^.

**Figure 10:**
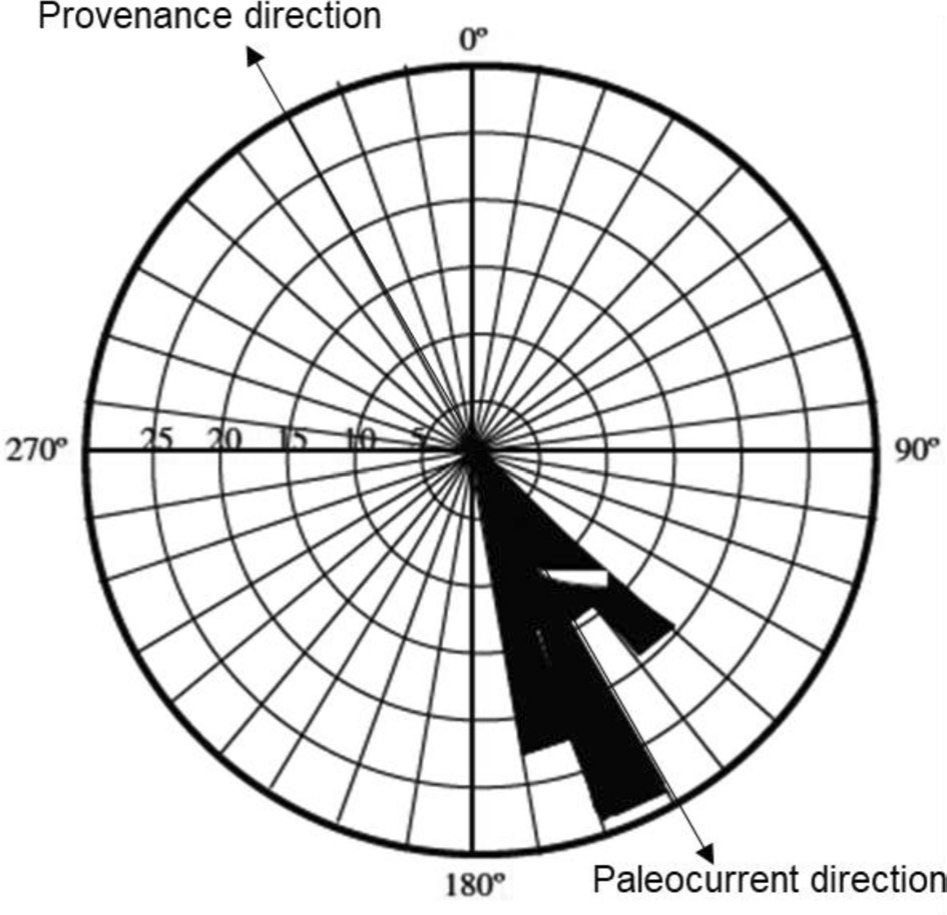
Rose diagram illustrating Paleocurrent and Provenance directions in the Ogwashi-Asaba Formation (Modified after^44^).

Paleocurrent data for the Abeokuta Group within the Eastern Dahomey Basin is not available but the most possible sediment source will be from the Western Nigeria basement located north of the basin (Fig. 1); whereas analyses of the Ogwashi-Asaba Formation within the Niger Delta Basin^44^ gave paleocurrent directions of approximately 150˚. Thus, the azimuth and the rose diagram suggest that the provenance direction of the sediment source for the Ogwashi-Asaba Formation is confined to the Northwest of the Niger Delta Basin (Fig. 10). This corresponds to sediment sources from the Western Nigeria and Northern Nigeria Basements (Fig. 1).

The basements are made up of the MGQC (e.g. the Archean Ibadan Grey Gneiss and Eburnean Ibadan Granite Gneiss) and Schist belt rocks (e.g. the Ife-Ilesa Schist belt rocks) within the Southwestern and Northern parts of Nigeria^13^. The Pan African Orogeny is well represented by the Older Granites dated at ca. 750–450 Ma^45^, which is associated with the MGQC and Schist belts as intrusions. Representation of the Kibaran orogeny is scarce in Nigeria. Hence, the little contributions from the Liberian and Kibaran orogenic rocks can probably be from the Banded Ibadan Grey Gneiss (Archean age) and minor mafic volcanics (Kibaran age) in the large Iseyin-Oyan River Schist Belt, respectively, both within the Western Nigeria Basement^13,46^.

Zircon ages from the Cretaceous soft Capim River kaolins (Brazil) indicated that 2.146 ± 0.023 Ga is the most representative age group with the highest number of grains followed by 1.881 ± 0.08 Ga^43^. Percentage contributions of the various detrital zircons from known orogenic events (Table 4) showed that the source sediments for the selected Cretaceous kaolins were mostly from areas related to the Transamazonian orogenic event in Brazil which corresponds to the Eburnean orogenic event in Nigeria except for the Nigerian Cretaceous Eruku kaolins with more contributions from areas related with the Pan African orogenic event. In addition, the Nigerian Cretaceous Lakiri kaolin deposit also had sediment contributions from areas related to the Pan African orogenic event in Nigeria which corresponds to the Braziliano orogenic event in Brazil. In contrast to the Cretaceous Nigerian kaolins (Lakiri and Eruku), the Brazilian Cretaceous soft Capim River kaolin deposit had considerable contributions from rocks with ages between 2000 and 1100 Ma, which correspond to the Kibaran orogenic event in Africa. Despite the similar time equivalence with respect to source rock ages and terranes, the available data is not enough to establish possible input of detritus from the West African Craton to South American Craton or vice versa.

## Conclusions

The studied detrital zircon grains were generally characterised by subhedral crystals within the Cretaceous kaolins which suggested relatively distant sediment sources. However, the detrital zircon grains within the Paleogene/Neogene were dominated by euhedral crystals indicating relatively closer sediment sources. The minimum provenance age estimates of rocks from which the Cretaceous–Paleogene/Neogene kaolins in the Eastern Dahomey and Niger Delta Basins in Nigeria were derived ranged from 553.2 ± 6.2 to 583.5 ± 2.0 Ma within the Ediacaran Period. Combined geochronological and paleocurrent information strongly demonstrate that the sediment sources of these kaolins were predominantly from rocks of Eburnean and Pan African ages with little contributions from rocks of Liberian and Kibaran ages. The Cretaceous kaolins originated from rocks in the Western Nigeria Basement, whereas the Paleogene/Neogene kaolins originated from both the Western and Northern Nigeria Basements.

## Supplementary Information

Supplementary Information.
